# In Vitro Lipophilic Antioxidant Capacity, Antidiabetic and Antibacterial Activity of Citrus Fruits Extracts from Aceh, Indonesia

**DOI:** 10.3390/antiox6010011

**Published:** 2017-02-03

**Authors:** Ernawita, Ruri Agung Wahyuono, Jana Hesse, Uta-Christina Hipler, Peter Elsner, Volker Böhm

**Affiliations:** 1 Institute of Nutrition, Friedrich Schiller University Jena, Dornburger Straße 25-29, Jena 07743, Germany; ernawita.ernawita@gmail.com; 2 Faculty of Education, Almuslim University, Jalan Almuslim No. 1, Bireun, Aceh 24261, Indonesia; 3 Department of Engineering Physics, Institut Teknologi Sepuluh Nopember Surabaya (ITS), Campus ITS Sukolilo, Surabaya 60111, Indonesia; ruri.tf014@gmail.com; 4 Department of Dermatology, University Medical Center Jena, Erfurter Straße 35, Jena 07743, Germany; Jana.Hesse@med.uni-jena.de (J.H.); Christina.Hipler@med.uni-jena.de (U.-C.H.); Peter.Elsner@med.uni-jena.de (P.E.)

**Keywords:** carotenoids, flavonoids, phenolic acids, L-TEAC, α-amylase, Microplate Laser Nephelometry

## Abstract

This study reports in vitro lipophilic antioxidant, inhibition of α-amylase and antibacterial activities of extracts of peel and pulp of citrus samples from Aceh, Indonesia. HPLC (high-performance liquid chromatography), phytochemical, and FTIR (fourier transform infrared) analysis detected carotenoids, flavonoids, phenolic acids and terpenoids, contributing to the biological potencies. Most peel and pulp extracts contained lutein and lower concentrations of zeaxanthin, α-carotene, β-carotene and β-cryptoxanthin. The extracts also contained flavanone glycosides (hesperidin, naringin and neohesperidin), flavonol (quercetin) and polymethoxylated flavones (sinensetin, tangeretin). L-TEAC (lipophilic trolox equivalent antioxidant capacity) test determined for peel extracts higher antioxidant capacity compared to pulp extracts. All extracts presented α-amylase inhibitory activity, pulp extracts showing stronger inhibitory activity compared to peel extracts. All extracts inhibited the growth of both gram (+) and gram (−) bacteria, with peel and pulp extracts of makin showing the strongest inhibitory activity. Therefore, local citrus species from Aceh are potential sources of beneficial compounds with possible health preventive effects.

## 1. Introduction

Plants are good sources of different phytochemical substances with diverse biological properties, which may be beneficial for human health. The human body naturally produces reactive oxygen species (ROS) such as superoxide anion radical, hydroxyl radical and hydrogen peroxide. Under normal conditions, enzymatic antioxidants (e.g. superoxide dismutase, glutathione peroxidase and catalase) and non-enzymatic antioxidants (e.g. carotenoids, vitamin C, vitamin E and polyphenols) can react with free radicals, resulting in less harmful products [1]. However, disturbance in the balance between these two, termed as oxidative stress, may cause degenerative diseases such as cancer, inflammation, and diabetes [2].

As one degenerative disease, type-2 diabetes is characterized by an insufficient amount of insulin circulating in the blood stream. α-Amylase and α-glucosidase are enzymes responsible for hydrolysis of starch to oligosaccharides and monosaccharides, consecutively, whose activity affects the blood glucose level. Many studies have been focused on the inhibition of α-amylase activity by food constituents, as disturbances in α-amylase activity resulted in a smaller amount of substrate being available to be converted by α-glucosidase, thus avoiding a sharp increase in the blood glucose level. Furthermore, interest in finding natural antibacterial compounds is encouraged by the increasing awareness of health-related risks of synthetic compounds, especially in food or nutraceutical preparations. In the food industry, antibacterial agents are used to extend food shelf-life. Nowadays, chemical preservatives are preferably replaced by natural antibacterial agents [3]. A wide array of compounds with antimicrobial activity can be found in plants, possessing an identical broad and safe activity, nonetheless with fewer side effects compared to antibiotics [4].

With a worldwide agricultural production up to 100 million metric tons per year, the use of various citrus species for bioactive compounds is appealing. Various citrus species have been traditionally used in India and Indonesia as remedies for diabetes, and scientific experiments have supported their uses [5,6]. The bioactivities of various citrus species extracts have also been reported, including antioxidant capacity of mandarin citrus [7] and hepatoprotective activity of pomelo and kaffir lime [8]. Essential oils of different citrus species (grapefruit, lemon, kumquat, orange, mandarin) also exhibited the growth inhibition of different strains of food-borne pathogen bacteria [9].

Interestingly, not only citrus pulps but also citrus peels, as a by-product, are a promising source of bioactive compounds, which are rich in carotenoids, phenolic acids, flavonoids, terpenoids and vitamin C. Main carotenoids reported for orange juice were (all-E)-β-cryptoxanthin, violaxanthin, antheraxanthin, (all-E)-β-carotene and lutein [10]. Different carotenoid contents may lead to different bioactivity, in particular antioxidant activity, as an earlier study [11] reported that structural characteristics such as the number of conjugated double bonds (c.d.b.) and the presence of functional groups on terminal rings of xanthophylls affect the α-TEAC-values (α-tocopherol equivalent antioxidant capacity) of carotenoids.

Flavonoids of citrus fruits consist of flavones, mainly concentrated in flavedo and flavanones, mainly located in albedo [12]. Previous studies reported that flavanones, flavones and polymethoxylated flavones (PMFs) exhibited beneficial effects, i.e., anti-inflammatory and hypocholesterolemic effects [13,14]. The flavanones, which are present in the metabolic process, are characterized by glycosides, including naringin, narirutin, hesperidin and neohesperidin and aglycones, including naringenin and hesperetin [15]. It has been reported that aglycone forms of citrus flavanones exhibited remarkably higher inhibitory activity than glycoside forms on baker’s yeast α-glucosidase enzyme assay [16]. Miyake et al., (2003) also reported that hydroxylated flavanones produced from hesperidin or naringin with Aspergillus have demonstrated higher antioxidant capacity than both glycoside and aglycone forms [17]. Furthermore, a systematic study has been undertaken revealing that the chemical structure of flavonoids, i.e., the unsaturated benzopyran ring, 3–OH, 4–CO, the linkage of the phenyl ring at the 3 position, and hydroxyl substitution at the phenyl ring, affects inhibition of α-glucosidase and α-amylase [18].

Aceh has different citrus species, and its tropical climate is in agreement for the growth of citrus plants. This study utilizes six (6) citrus species which some of them are believed to be found only in Aceh (mentui, kruet mameh, makin, calung) and jeruk nipis and jeruk purut. The use of these citrus plants has been previously described [19]. Nevertheless, still very limited scientific data is available on the biological properties of these citrus plants. The present work deals with the analysis of lipophilic antioxidant capacity, α-amylase inhibitory activity and antibacterial activity of various citrus fruits originally from Aceh, Indonesia. To the best of our knowledge, there are no data available on in vitro lipophilic antioxidant capacity, α-amylase inhibition and antibacterial activity for the citrus fruits from Aceh used in this study. Our previous work exhibited that one of the indigenous citrus fruits from Aceh is rich in carotenoids as well as in flavonoids and shows promising bioactivity [19]. This study, in addition, sheds light on the initial investigations of the effects which different carotenoids, phenolic acids and flavonoids contained in various citrus extracts have regarding their antioxidant, antidiabetic and antibacterial activity.

## 2. Materials and Methods

### 2.1. Chemicals

All solvents used were of HPLC grade. Reagents used were of analytical grade. Carotenoid standards were purchased from CaroteNature (Ostermundigen, Switzerland). Polyphenol standards were from Sigma-Aldrich (Taufkirchen, Germany) and Extrasynthese (Genay, France). Staphylococcus aureus ATCC 6538 and Klebsiella pneumoniae ATCC 4352 were purchased from the DSMZ (Deutsche Sammlung von Mikroorganismen und Zellkulturen, Braunschweig, Germany).

### 2.2. Plant Materials

Citrus samples studied here were calung, makin, mentui, kruet mameh, jeruk nipis (*C. aurantiifolia*) and jeruk purut (*C. hystrix*, kaffir lime), for more details on the samples see [19].

### 2.3. Carotenoid Extractions

Citrus carotenoids were extracted using a previously described method [20]. Briefly, ca. 500 mg of sample were extracted with methanol:tetrahydrofuran (1 + 1, v/v) using an ultra turrax until the extraction solution was colourless.

### 2.4. Polyphenol Extractions

Prior to extraction, citrus samples were hydrolysed using hydrochloric acid and sodium hydroxide in order to release the phenolic compounds from the sample matrix. Hydrolysed samples were extracted with 50% aqueous ethanol. These extracts were then subjected to further experiments. For HPLC and LC-MS (liquid chromatography mass spectrometry) analysis, (−)-epicatechin was used as internal standard to calculate the recovery rate. All extractions were performed in triplicate.

### 2.5. Phytochemical Analysis

The following phytochemical analysis was conducted qualitatively according to methods described by [4].

#### 2.5.1. Phenolic Test

Citrus extracts were dissolved in methanol (MeOH) and 5% ferric chloride solution was added dropwise. The apparent dark bluish colour of the resulting solution indicated the presence of phenolic compounds.

#### 2.5.2. Flavonoid Test

Citrus extracts were dissolved in MeOH and a few drops of 10% aqueous NaOH were added to obtain an intense yellow colour. The conversion into a colourless solution upon addition of diluted HCl indicated the presence of flavonoids.

#### 2.5.3. Terpenoids (Salkowski Test)

Citrus extracts (0.5 mg) were dissolved in a mixture of concentrated H_2_SO_4_ and chloroform (equal volumes) to form a layer. The formation of reddish brown colour at the interface indicated the presence of terpenoids.

#### 2.5.4. Alkaloid Test (Wagner Test)

Citrus extracts (50 mg) were stirred with a few mL of diluted HCl and filtered. A few drops of Wagner’s reagent were added at the side of the test tube. The formation of reddish brown precipitates showed the presence of alkaloids.

#### 2.5.5. Cardiac Glycoside Test

Citrus extracts (50 mg) were diluted with 2 mL of glacial acetic acid containing one drop of 5% ferric chloride solution, followed by addition of 1 mL of concentrated H_2_SO_4_. A brown ring at the interface indicated the presence of cardenolide deoxy sugar. A violet ring below the brown ring and a greenish ring in the acetic acid layer indicated the presence of cardiac glycoside.

### 2.6. HPLC Analysis

#### 2.6.1. Carotenoid Analysis

Carotenoid contents of the samples were analysed using a C_30_ HPLC column according to a method previously described by [21]. Briefly, analysis was performed on HPLC apparatus with diode array detector at 450 nm (Merck Hitachi, Darmstadt, Germany) using a Develosil RP-aqueous (250 mm × 4.6 mm, 5 µm) C_30_ column (Phenomenex, Aschaffenburg, Germany) at 13 ± 1 °C and a gradient of methanol and methyl tert-butyl ether as mobile phase.

#### 2.6.2. Polyphenol Analysis

Citrus polyphenols were quantified using an HPLC system (Merck) equipped with Biotech DEGASi compact 4 channels degasser, L-7200 autosampler, L-7100 pump, L-7450A diode array detector (DAD), and a Luna C18(2) column (250 × 4.60 mm, 5 µM) (Phenomenex, Aschaffenburg, Germany). The method was used as described by Abad-Garcia et al. [22] with a slight modification. The mobile phase was composed of H_2_O with 0.5% acetic acid (solvent A) and methanol (solvent B). The elution conditions were: 0–2 min (100% A), 2–6 min (100%–85% A), 6–12 min (85% A), 12–17 min (85%–80% A), 17–35 min (80% A), 35–90 min (80%–65% A), 90–132 min (65% A), followed by washing and reconditioning periods. Column temperature was maintained at 30 ± 1 °C with a flow rate of 0.8 ml/min. Injection volume was 50 µL. Analysis was performed in triplicate. Samples integration was set at 265 nm (hydroxybenzoic acid), 280 nm (flavanones), 320 nm (flavones and hydroxycinnamic acid) and 370 nm (flavonols and coumarins).

### 2.7. FTIR Analysis

Fourier transform infrared (FTIR) spectroscopies were conducted as described in [19]. Following the previously reported IR study [23], interpretation and analysis neglected the spectral features at the wave number range of > 3500 cm^−1^ due to the incurrence of a broad peak of hydroxyl (–OH) stretching vibration overlapping with other bands. In order to get the quantitative estimation of flavonoid, phenolic acid or carotenoid contents, a particle swarm optimization was used for fitting the IR (infrared) spectra [24] with sum of reference spectra of suggested flavonoid, phenolic and carotenoid contents. The optimization used a number of swarmed particles spanning between 500 and 1000 with up to 1000 iterations.

### 2.8. Antioxidant Capacity

Lipophilic antioxidant capacity was measured using α-TEAC method according to Müller and co-workers [11] with a slight modification as can be seen in [19]. Briefly, citrus carotenoids extracts were dried (under nitrogen gas) and dissolved again in methanol. Carotenoids were extracted using petroleum ether. These solutions were dried and re-dissolved in hexane. Antioxidant capacity was measured determining the loss of colour of an ABTS^•+^ solution using α-tocopherol (4–120 µmol/L) as reference compound. Results are given as µmol α-TE (α-tocopherol equivalents)/100 g.

### 2.9. Inhibition of α-Amylase Activity

Inhibition of α-amylase activity test was performed according to Apostolidis and co-workers [25] with slight modification as described in [19]. Briefly, an aliquot of extract (in 5% Tween-40 + 5% DMSO (dimethyl sulphoxide), dissolved in pH 6.9 sodium phosphate buffer solution) were mixed stepwise (with incubations in between) with α-amylase in sodium phosphate buffer, starch solution and 3,5-dinitrosalicylic acid colour reagent. Seven (7) dilutions of test solutions were prepared for each analysis. The absorbance was measured at 540 nm. Acarbose was used as positive control. Results are calculated by generating a dose response curve through linear regression using Origin version 8 software (OriginLab Corporation, Northampton, MA, USA). The results are given as IC_50_ (half maximal inhibitory concentration).

### 2.10. Antibacterial Activity

Antibacterial activity was determined according to methods previously described [19].

#### 2.10.1. Extract Preparation

Citrus extracts were prepared as previously described. The resulting extracts were dried completely under nitrogen gas and stored at −20 °C prior to use. Samples were then prepared by diluting in 0.75% Tween-40 and 9.25% DMSO and sterile TSB (tryptic soy broth) to make up to 100%.

#### 2.10.2. Preparation of Bacterial Suspensions

Cultured bacteria were prepared one day prior to the test as described in [19].

#### 2.10.3. Microplate Laser Nephelometry

Bacterial growth was monitored by Microplate Laser Nephelometry (MLN) method as described in [19]. Serial dilutions were prepared according to Finger and co-workers [26]. Shortly, five different concentrations were prepared for each extract for each independent experiment. Sample reading was performed in the microplate laser nephelometer for 24 h at 37 °C. A growth curve was then built using the turbidity intensity, and the area under the curve was determined. Percentages of the viable bacteria were calculated to prepare a dose response curve from which the IC_50_ (half maximal inhibitory concentration) values of citrus samples were calculated. Sample wells which did not show any turbidity were sub-cultured in Columbia agar plates and incubated for 18 h at 37 °C. Samples which then did not show colony growth were considered to have bactericide activity, while bacteriostatic activity is described when bacterial colony growth was present in sub-cultured medium.

### 2.11. Statistical Analysis

The values are expressed as mean ± standard deviation (SD). Statistical difference was determined using SPSS 22 (Statistical Package for the Social Sciences, Chicago, IL, USA) one-way ANOVA followed by Tukey’s test for post hoc analysis. *p* < 0.05 was considered as significant.

## 3. Results and Discussion

### 3.1. HPLC and FTIR Analysis

**Figure 1:**
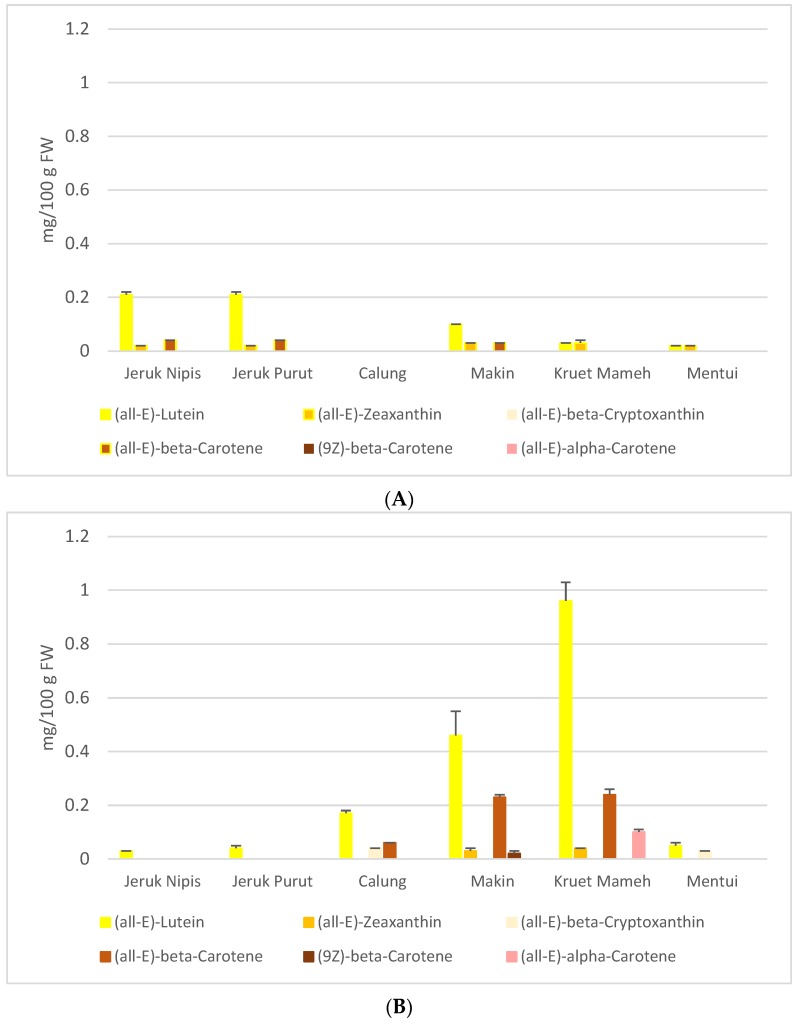
Carotenoid composition of peel (**A**) and pulp (**B**) of citrus fruits from Aceh (adapted from Ernawita et al., 2016 [19]).

In recent studies [19, unpublished data], the carotenoid contents in MeOH/THF (1 + 1, v/v extracts and contents of some flavonoids and polyphenolics in EtOH/H2O (1 + 1, v/v) extracts have been measured by either HPLC or HPLC-MS (Figure 1 and Table 1). (all-E)-Lutein and (all-E)-zeaxanthin are carotenoids identified in almost all citrus MeOH/THF extracts (Figure 1), while flavanones, mainly hesperidin, neohesperidin and naringin, are the main phenolic compounds having been identified (Table 1).

**Figure 2:**
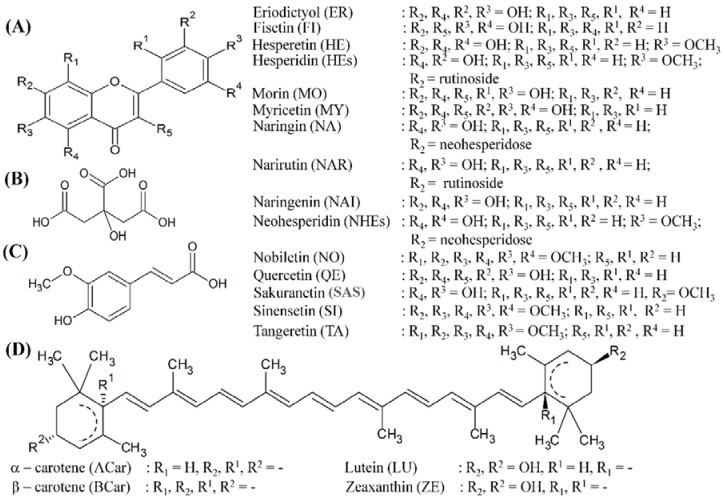
Schematic representation of reference (**A**) flavonoids, phenolics: (**B**) citric acid and (**C**) ferulic acid, and (**D**) carotenoids in IR analysis.

**Figure 3:**
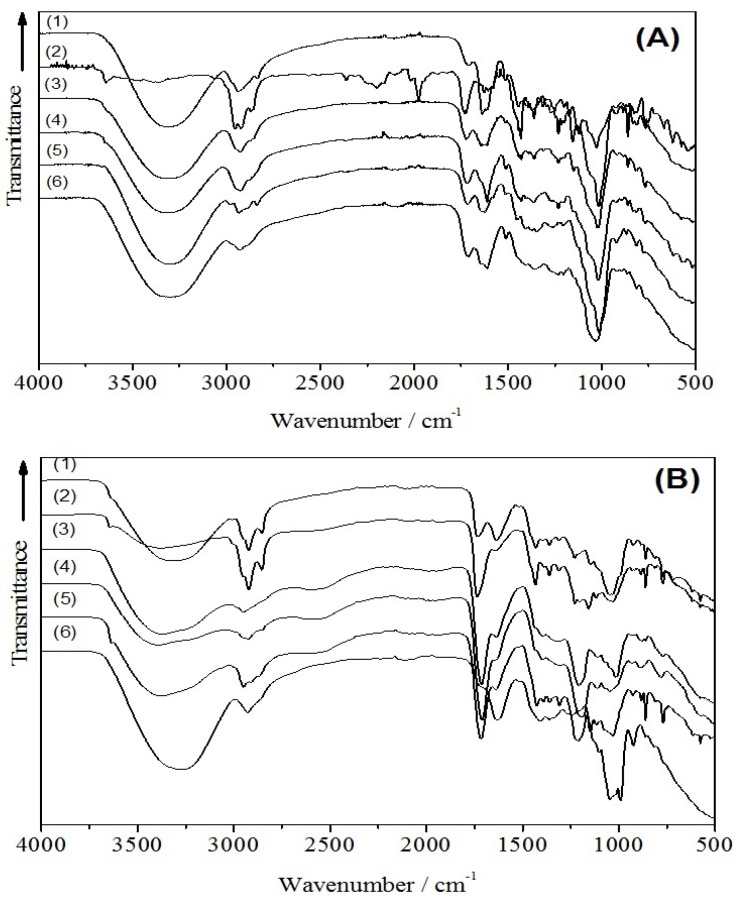
FTIR spectra of various citrus species extracted from the peels (**A**) and the pulps (**B**).The number indicates the citrus species in the following order: (1) calung, (2) makin, (3) mentui, (4) jeruk nipis, (5) jeruk purut, (6) kruet mameh.

In addition, infrared technique was used in this study for the identification of other flavonoids and carotenoids in the citrus extracts. By using the virtue of fundamental and harmonic vibrational features, this technique enabled the estimation of either flavonoid, phenolic acid or carotenoid contents contained in a sample. Considering previous findings of flavonoid, phenolic and carotenoid contents in the citrus extracts, the flavonoids and its derivatives, e.g. flavonols and either flavanones or flavanone-glycosides, phenolic acids, e.g. citric and ferulic acid, and carotenoids, e.g. lutein, zeaxanthin, α- and β-carotene, were considered as the reference spectra. The schematic chemical structures of the reference flavonoid, phenolic and carotenoid are shown in Figure 2. The infrared transmission spectra of various citrus extracts of both peel and pulp are depicted in Figure 3A,B.

The general assignments of infrared spectra are the following: The infrared band at 3500–3200 cm^−1^ could be assigned to O–H stretching vibration of either macromolecular compounds or absorbed water. The infrared band at 3000–2800 cm^−1^ was attributed to C–H stretching vibration of methyl, methoxy and methylene groups originated from two moieties of flavonoid, i.e., benzopyran and phenyl group. The carbonyl (C=O) stretching vibration of the carboxyl group (COO) was indicated by the spectral peak at 1740–1725 cm^−1^. The infrared bands around 1638 cm^−1^ were ascribed as the C=C stretching that is attributed to the presence of aromatic or benzene rings. The vibrational bands at around 1430–1455 cm^−1^ were ascribed to aliphatic and aromatic (C–H) groups in the plane deformation vibrations of methyl, methylene and methoxy groups. The bands in the range 1300–1000 cm^−1^ were due to the C–O stretching vibration of carboxylic acids and alcohols. The 1130–1150 cm^−1^ bands originated from vibration of C–O–C and O–H of polysaccharides. Spectral features at the wavenumber of 900 cm^-1^ or less were assigned to the finger print zone. The detailed IR analysis and fitting results are summarized in Table 2. The IR analysis was in good agreement with our previous HPLC and LC-MS analysis [19]. In addition, other flavonoids contained in the citrus extracts studied here were identified (see Table 1). The results show that citrus peels contain more diverse flavonoids than citrus pulps, including numerous flavanones, flavone *O*-glycosides and the polymethoxylated flavones (PMFs). PMFs are mainly obtained in citrus peels as highly methoxylated flavone aglycones.

### 3.2. Antioxidant Capacity of Citrus Samples

L-TEAC test is used to determine the lipophilic antioxidant capacity of lipophilic compounds. Decrease in the absorbance of ABTS radical solutions indicates the presence of antioxidant properties in the tested samples and the changes can be monitored in a spectrophotometer. In most cases, antioxidant capacity of citrus has been mainly attributed to the polyphenols of citrus, mainly from the class of flavanones such as hesperidin, neohesperidin, naringin, narirutin and neoeriocitrin [27], vitamin C, vitamin E and phenolic acids [28].

**Figure 4:**
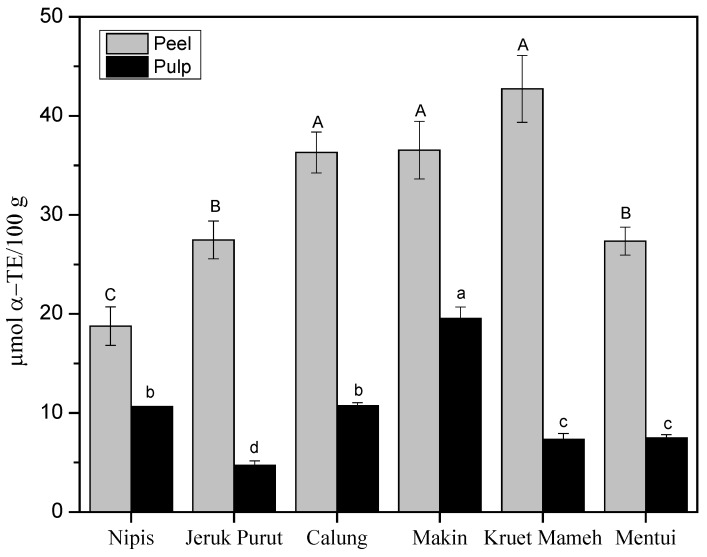
L-TEAC values (µmol α-TE/100 g) of 50% MeOH/THF extracts of peels and pulps of citrus samples. Values are expressed as mean ± SD (*n* = 3). Bars with different letters are significantly different (One Way ANOVA, Tukey HSD test, *p* < 0.05). Capital (A,B,C) and small (a,b,c) letters indicate the comparison done in its respective categories.

L-TEAC antioxidant capacity test (Figure 4) showed that the peels had higher antioxidant capacity compared to the pulps. L-TEAC values of peels varied from 18.8 ± 2.0 to 42.7 ± 3.4 µmol α-TE/100 g. The peel of kruet mameh showed the highest antioxidant capacity, followed by the peels of calung and makin, but there was no significant difference among these three samples. Peel of jeruk nipis (18.8 ± 2.0 µmol α-TE/100 g) showed the lowest antioxidant capacity. For pulp extracts, makin showed the highest antioxidant capacity (19.5 ± 1.2 µmol α-TE/100 g), followed by the pulps of calung and jeruk nipis (10.7 ± 0.3 and 10.6 ± 0.0 µmol α-TE/100 g).

Antioxidant capacity of peel extracts might stem from the presence of carotenoids and flavonoids. On the one hand, a previous study [11] reported that the order of α-TEAC-values of carotenoids is influenced by the number of c.d.b. and the functional groups on terminal rings of xanthophylls, and the α-TEAC values are descending in the order of (all-E)-lycopene > (all-E)-α-carotene > (all-E)-β-cryptoxanthin > (all-E)-β-carotene > (all-E)-lutein > (all-E)-zeaxanthin. Peel extract of jeruk purut contained (all-E)-lutein, (all-E)-β-carotene, (all-E)-α-carotene and (all-E)-zeaxanthin. Peel extracts of makin and calung contained (all-E)-lutein, (all-E)-zeaxanthin and (all-E)-β-carotene in lower concentrations than those of kruet mameh (Figure 1). Thus, higher antioxidant capacity was shown for peel extracts of kruet mameh compared to other peel extracts. In jeruk nipis, only (all-E)-lutein was quantified in the peel extracts, thus contributing to its low antioxidant capacity. On the other hand, higher antioxidant capacity in citrus peels were supported by the facts that they contained more diverse flavonoids than citrus pulps, including numerous flavanones, flavone *O*-glycosides, and PMFs, hence might increase the scavenging capacity of peroxyl and hydroxyl radicals [11].

Pulp extracts in general had lower concentrations of carotenoids, thus lower antioxidant capacities were obtained in pulps of citrus samples studied here. Pulp extracts of makin and jeruk nipis contained (all-E)-lutein, (all-E)-zeaxanthin and (all-E)-β-carotene. Interestingly, no carotenoids were determined in the pulp extracts of calung [19]. Hence, its antioxidant capacity might be caused by flavonoids and phenolic acids as observed in IR analysis. It should be noted that citrus extracts studied here were prepared in a mixture of methanol and tetrahydrofuran [11,20]. However, this solvent mixture enables extraction not only of carotenoids but also of other compounds, varying from polyphenols (more polar compounds) to terpenoids (less polar compounds). Therefore, phytochemical tests were conducted to detect the presence of other compounds (Table 3). Phytochemistry test showed that peels and pulps of citrus samples contained phenolics, flavonoids and terpenoids, which is in good agreement with previous findings [29] and might also contribute to their antioxidant capacity as previously discussed.

### 3.3. In vitro α-Amylase Activity of Peels and Pulps of Citrus Fruits

**Figure 5:**
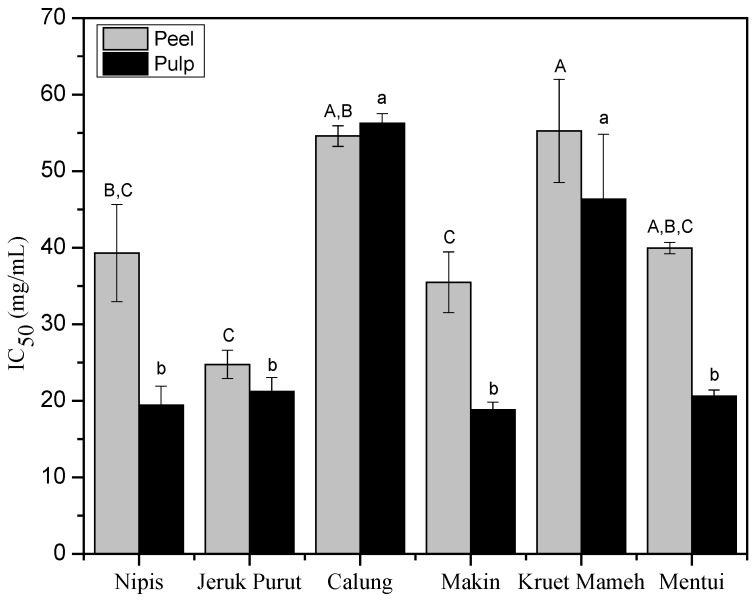
α-Amylase inhibitory activity (IC_50_: mg/mL) of 50% MeOH/THF extracts of peels and pulps of citrus samples. Values are expressed as mean ± SD (*n* = 3). Bars with different letters are significantly different (One Way ANOVA, Tukey HSD test, *p* < 0.05). Capital (A,B,C) and small (a,b,c) letters indicate the comparison done in its respective categories.

In vitro α-amylase inhibitory activity of 50% MeOH/THF extracts of peel and pulp of citrus samples (Figure 5) showed moderate inhibitory activity compared to acarbose (IC_50_ < 0.04 mg/mL). The pulp, in general, had greater α-amylase inhibition activity compared to the peel. Additionally, there was no significant difference among IC_50_ of pulp extracts of jeruk nipis, makin, jeruk purut and mentui. Pulp extracts of makin and jeruk nipis showed the lowest IC_50_ (18.8 ± 1.0 mg/mL and 19.4 ± 2.5 mg/mL, respectively), while calung showed the highest IC_50_ (56.2 ± 1.3 mg/mL). For peels, jeruk purut showed the lowest IC_50_ (24.8 ± 1.8 mg/mL), followed by makin (35.5 ± 4.0 mg/mL). The peels of calung and kruet mameh showed the lowest inhibitory activity (IC_50_: 54.6 ± 1.3 mg/mL and 55.2 ± 6.7 mg/mL, respectively).

Potency of jeruk purut juice as α-amylase inhibitor has been previously reported as comparable with acarbose [30], while a study on α-amylase inhibitory activity of other citrus samples is not found. Different natural compounds have been reported to inhibit α-amylase activity. A recent study [31] proposed saponins, steroids and terpenoids as compounds being responsible for α-amylase inhibitory activity of *C. microptera*. Tannins, anthocyanins and polyphenols were reported to show a different degree of inhibition on α-amylase activity depending on the structural configuration. Specific binding site, structural conformation and competitive inhibition have been proposed as mechanisms to inhibit α-amylase activity [32]. Sinensetin isolated from *Orthosiphon stamineus* was also reported to inhibit α-amylase activity [33]. FTIR analysis (Table 2) presented different compounds being present in citrus peel and pulp extracts which might contribute to α-amylase inhibitory activity. In one genus of plant, it is often observed that similar compounds are present, but the concentration (or composition) may vary. Citric acid is often found in abundance in pulp of citrus, affecting its pH. FTIR analysis (Table 2) showed that citric acid concentration varied in pulp extracts, and it is interesting to note that jeruk calung pulp extract had the lowest citric acid concentration and showed the lowest inhibitory activity. In peel extracts, being not rich in citric acid, differences in pH levels are believed to be not significant. Interactions between different compounds in peel extracts more likely contribute to its α-amylase inhibitory activity. Thus, it will be interesting to test the effects of those identified compounds, either individually or in combination, on its in vitro α-amylase inhibitory activity in the future.

### 3.4. Antibacterial Activity of Peel and Pulp of Various Citrus from Aceh

Table 4 summarizes the antibacterial activity of peel and pulp extracts of citrus samples tested here. All citrus extracts in general inhibited the growth of bacteria and the activity differed among species. Citrus extracts were slightly more potent against gram (+) compared to gram (−) bacteria. Peel extracts exhibited slightly better activity compared to the corresponding pulp extracts. Interestingly, pulp extracts were more potent to rapidly kill the tested bacteria, while peel extracts mostly inhibited or delayed the growth of tested bacteria. The results were corroborated by a previous report on antibacterial activity of peel and pulp of jeruk purut against *S. aureus* and *K. pneumoniae* [30]. Antibacterial activity of citrus extracts could originate from the flavonoid contents, particularly polymethoxylated flavones (PMFs), i.e., sinensetin, nobiletin and tangeretin. Their activities on inhibiting the growth of bacteria and fungi have been reported [34]. Yi and co-workers [35] reported an IC_50_ of 1.6 mg/mL for nobiletin and tangeretin against *S. aureus*, while synergistic activities of different polymethoxylated flavones on inhibiting the growth of fungi have also been reported. Direct contact of polymethoxylated flavones led to damaged bacterial cell walls which were indicated by flowing Na^+^ and K^+^ from inner cell wall to outer cell wall. These mechanisms have been proposed as antibacterial mechanisms of polymethoxylated flavones [36].

Citrus fruits contain relatively higher citric acid contents compared to other fruits. Citric acid has been reported to be the main organic acid identified in juices of citrus fruits. Citric acid activity on inhibiting the growth of Vibrio bacteria has been shown. Citric acid contributed to pH of fruit juices, and juices with lower pH had better antibacterial activity compared to the same juice with modified higher pH [36].

From these initial data of biological potencies of local citrus fruits from Aceh, some facts are worth to notice. Crude extracts of peel and pulp of citrus samples are comprised of different classes of compounds (Table 2) which are metabolized differently in the body. Hydrophilic compounds such as polyphenols are rapidly metabolized and the excretion of the compounds from the body generally is achieved in less than 24 hours. Peak concentrations of flavanones in plasma are reported between 4 and 7 h. Thus, the replacement of those compounds by continuous intake is necessary. Understanding the metabolism of flavanones as the main flavonoids of citrus fruits is particularly important. Flavanone glycosides will undergo hydrolysis, mainly in the colon, due to specific enzymes available in colon microflora. There, the resulting aglycone is then absorbed and metabolized by the body [37]. In addition, as flavanones are generally less polar compared to other flavonoids, their initial form and solubility are also important since the bioavailability of flavanones is also affected by them. In one human intervention study, a positive correlation has been found between solubility of flavanones and its excretion rate [38]. On the other hand, lipophilic compounds, i.e., carotenoids, are of high importance due to the nature of its metabolisms in the body. Unlike hydrophilic compounds, carotenoids are stored in the body, particularly in liver and fat, and can be metabolized for a much longer period. The ability to store the potent compounds will be preferable for diseases prevention, or as a source of the essential nutrient retinol [39]. Of several carotenoids identified in peel and pulp of citrus extracts, β-cryptoxanthin, which was detected in some of the citrus sample extracts, is remarkably interesting due to its high bioavailability in human metabolism. Different studies also showed that β-cryptoxanthin has a better bioavailability compared to β-carotene [40]. Moreover, β-cryptoxanthin is a well-known vitamin A precursor, and has been linked with the ability to delay osteoporosis and to prevent certain types of cancers [40]. In a country with a substandard health care system as Indonesia, knowledge of beneficial nutrition with preventive potency will be very useful. Therefore, further studies on different aspects affecting concentrations and bioavailability of carotenoids in citrus fruits and other local fruits are needed. Factors such as seasonal variation, geographical difference, effect of food preparation will have to be taken into consideration.

## 4. Conclusions

Our results show that peel and pulp extracts of indigenous citrus species from Aceh in general are comprised of diverse carotenoids, phenolic acids and flavonoids. Overall, peel and pulp extracts of kruet mameh and makin were rich in carotenoids (lutein and β-carotene), flavonoids (hesperidin, naringin, tangeretin) and phenolic acids, and showed promising in vitro antioxidant, antidiabetic and antibacterial activities. Additionally, the consumption of whole fruits, rather than its single compounds, is considered to be more beneficial for health due to complex mixtures of substances present and possible interactions.

## Figures and Tables

**Table 1:** Polyphenols in peel and pulp of citrus samples from Aceh. Results are expressed as mean ± SD (*n* = 3).

No.	Compound	Class	WL (nm)	RT (min)	Max. WL (nm)	Concentration (mg/100 g FW) in											
						Jeruk Nipis		Jeruk Purut		Calung		Makin		Kruet Mameh		Mentui	
						Peel	Pulp	Peel	Pulp	Peel	Pulp	Peel	Pulp	Peel	Pulp	Peel	Pulp
1	Gallic acid	HBA	254	11.4 ± 0.0	273	11 ± 2	n.d.	8.3 ± 0.3	1.5 ± 0.1	14 ± 2	2.3 ± 0.1	13 ± 3	2.6 ± 0.0	7.2 ± 0.4	4.1 ± 2.1	7.8 ± 0.9	0.9 ± 0.0
2	Caffeic acid	HCA	320	30.5 ± 0.0	297, 323	4.6 ± 2.2	n.d.	n.d.	n.d.	13 ± 1	1.5 ± 0.0	4.8 ± 0.2	n.d.	22 ± 0	2.3 ± 0.3	n.d.	n.d.
3	Ferulic acid	HCA	320	55.5 ± 0.1	290, 320	2.8 ± 0.1	n.d.	3.4 ± 0.2	n.d.	53 ± 1	3.4 ± 0.1	3.8 ± 0.7	n.d.	24 ± 1	3.4 ± 1.0	2.0 ± 0.2	n.d.
4	Narirutin	FVN	280	81.3 ± 0.0	274	n.d.	n.d.	n.d.	n.d.	n.d.	n.d.	n.d.	n.d.	n.d.	n.d.	n.d.	n.d.
5	Naringin	FVN	280	86.4 ± 0.3	286, 334	n.d.	n.d.	n.d.	n.d.	952 ± 63	271 ± 10	n.d.	n.d.	n.d.	n.d.	n.d.	n.d.
6	Hesperidin	FVN	280	91.0 ± 0.1	285, 333	299 ± 17	71 ± 1	130 ± 5	74 ± 7	n.d.	n.d.	232 ± 10	103 ± 11	1422 ± 36	338 ± 7	29 ± 1	23 ± 0
7	Neohesperidin	FVN	280	95.5 ± 0.1	285, 333	n.d.	n.d.	150 ± 4	75 ± 4	1137 ± 56	158 ± 1	233 ± 14	123 ± 15	n.d.	n.d.	114 ± 8	51 ± 2
8	Sinensetin	FLV	320	150.8 ± 0.0	242, 266 *, 332	0.3 ± 0.1	n.d.	n.d.	n.d.	2.7 ± 1.5	0.2 ± 0.0	1.6 ± 0.1	n.d.	3.0 ± 0.3	n.d.	n.d.	n.d.
9	Nobiletin	FLV	320	152.9 ± 0.0	251, 271, 334	0.4 ± 0	n.d.	n.d.	n.d.	24 ± 1	n.d.	7.8 ± 0.4	n.d.	39 ± 1	n.d.	n.d.	n.d.
10	Tangeretin	FLV	320	154.7 ± 0.0	272, 334	2.4 ± 0.1	n.d.	n.d.	n.d.	7.7 ± 0.4	0.9 ± 0.0	4.7 ± 0.1	n.d.	5.1 ± 0.2	n.d.	n.d.	n.d.

HBA: hydroxybenzoic acids, HCA: hydroxycinnamic acids, FVN: flavanones, FLV: flavones, n.d.: not detected, FW: fresh weight, WL: wave length, RT: retention time, * second peak.

**Table 2:** Infrared bands in the spectral region of 3500–500 cm^−1^ of various citrus extracts and the fitting results to the reference flavonoids, phenolic acids and carotenoids.

Functional group	Peel						Pulp					
	Calung	Makin	Mentui	Jeruk Nipis	Jeruk Purut	Kruet Mameh	Calung	Makin	Mentui	Jeruk Nipis	Jeruk Purut	Kruet Mameh
O–H stretching	3320	3644.1; 3358.6	3294.7	3315.6	3300.1	3311.1	3000.2	3642.2; 3371.9	3377.3	3397	3635.9; 3389.2	3260.7
CH_2_ vibration	2937.3; 2831.7	2952.9; 2922.4	2925.1	2925.5	2977.3; 2831.7	2930	2922.9; 2854.1	2922.8; 2854	2949.8	2925.5; 2844.3	2951.3	2927.8
COO stretching	1704.5	1725.5	1720.3	1719.2	1716.3	1710.3	1736.1	1734.4	1713	1809.9	1716.4	
C=O stretching	1636	1625.7; 1605.1	1638.6	1611	1623.2	1610.1	1637.1	1638	1628.5		1634.8	1628.6
C=C vibration	1511.4; 1441.7; 1400.5	1547.4; 1441.1; 1430.7	1429.7	1511.4; 1429.7	1511.4; 1444.9	1510	1429.8	1430.5; 1389.8	1394.2	1397.4	1428.1; 1388.8	1406.1
CH bending	1369.4; 1271.5	1360.4; 1314.1	1359.5	1362.8	1347.5	1355.3	1359.8; 1305.5	1360.8; 1313.4	1315.1		1357.8; 1308.4	1362.5
COO vibration	1197.9; 1173.4	1230.8; 1214.3; 1155.4	1230.3	1230.5	1204.2	1204.0	1230; 1144.1	1230.0; 1214.2; 1191.6	1208.3	1196.9; 1108.1	1213.3; 1147.2; 1118.9	1232.7; 1197.9; 1102.9
C–O–C vibration	1016.6	1026.7	1021.7	1018.3	1015	1030.8	1050.1	1025.6	1015.4	1047.5	1025.1	1046.9
Finger print zone	919.3; <812.7	919.2; <885.9	860.27; <767.8	922.4; <887.6	916.1; <862.3	<920	925.1; <860.3	935.1; <885.6	881.9	881.42	929.1; <884.3	991.3; 924
Flavonoid, phenolic acid and carotenoid contents * (value in brackets indicate percentage)	BCar (16), CA (0.6), ER (7), FA (4), FI (2), HE (7), HEs (5), LU (4), NA (14), NAI (4), NHEs (5), NO (0.6), QE (7), SA (2), SI (1), TA (16), ZE (4)	BCar (13), CA (4), FA (1), HE (6), HEs (6), LU (10), NAI (7), NHEs (12), SI (0.5), TA (42)	BCar (7), CA (4), FA (7), FI (2), HEs (8), LU (20), NA (10), NAI (6), NAR (3), NHEs (4), MO (2), MY (2), QE (7), SA (1), TA (15), ZE (6)	CA (4), FA (0.5), HEs (11), LU (13) NA (12), NO (5.5), QE (14), SA (18), SI (1), TA (21)	CA (2.7), FA (2.7), HEs (22.4), LU (7), NA (20.3), NHEs (11.7), NO (4.3), SA (3), TA (25.6), ZE (0.3)	ACar (2.5), BCar (1.5), CA (4.7), ER (3.5), FA (2.4), FI (15), HEs (2), LU (11.5), NA (13), NAI (1), NAR (4), QE (9.8), SA (6), SI (2), TA (21), ZE (0.2)	ACar (4), CA (5), ER (13), FA (6), HE (4), HEs (1) LU (16), NA (11), NAI (3), NHEs (2.3), QE (5), SA (5), SI (2), TA (22)	BCar (12.7), CA (12), HEs (12), LU (12), MY (5.5), NA (3), NAI (5.5), NAR (5.5), NHEs (14), TA (25)	CA (16), FA (10), FI (3.3), HEs (2), LU (5), NA (13), NHEs (18), NO (1), SA (2.3), SI (4), TA (16), ZE (9.5)	BCar (4.8), CA (15.6), FA (6.7), HE (3), HEs (5), LU (6.8), MO (3), QE (1.4), SA (31), TA (19.6), ZE (2)	BCar (7), CA (13.8), ER (5), FA (8), HEs (4.4), LU (11.7), NA (8.3), NHEs (20.4), NO (5), MY (3.6), QE (4.9), TA (7.7)	CA (6.6), FA (3), FI (3.3), HE (3), HEs (11), LU (4), MO (3.3), NA (17.2), NHEs (2), QE (6.7), SA (3.7), SI (12), TA (20.2), ZE (4)

* ACar = α-carotene, BCar = β-carotene, CA = citric acid, ER = eriodictyol, FA = ferulic acid, FI = fisetin, HE = hesperetin, HEs = hesperidin, LU = lutein, MY = myricetin, MO = morin, NA = naringin, NAI = naringenin, NAR = narirutin, NHEs = neohesperidin, NO = nobiletin, QE = quercetin, SA = sakuranetin, SI = sinensetin, TA = tangeretin, ZE = zeaxanthin.

**Table 3:** Phytochemical test results of MeOH/THF extracts of citrus samples.

Sample	Phenolics		Flavonoids		Terpenoid		Alkaloid		Cardiac glycoside	
	Peel	Pulp	Peel	Pulp	Peel	Pulp	Peel	Pulp	Peel	Pulp
Jeruk Nipis	+	+	+	+	+	+	−	−	+	+
Jeruk Purut	+	+	+	+	+	+	−	−	+	+
Calung	+	+	+	+	+	+	+	−	+	+
Makin	+	+	+	+	+	+	−	−	+	+
Kruet Mameh	+	−	+	+	+	+	−	−	+	+
Mentui	+	+	+	+	+	+	−	−	+	+

+ = present, − = not present.

**Table 4:** Antibacterial activities (IC_50_: mg/mL) of peels and pulps of citrus samples from Aceh. Different letters (a,b) indicate a significant difference within the column (One Way ANOVA, post hoc Tukey’s test, *p* < 0.05).

Sample	Peel				Pulp			
	*K. pneumoniae*	Activity	*S. aureus*	Activity	*K. pneumoniae*	Activity	*S. aureus*	Activity
Jeruk Nipis	4.2 ± 1.8a	Bactericide	3.5 ±1.4a,b	Bacteriostatic	4.1 ± 0.4a,b	Bactericide	3.1 ± 0.6a,b	Bactericide
Jeruk Purut	4.6 ± 1.2a	Bacteriostatic	4.8 ± 1.7a,b	Bactericide	5.5 ± 1.1a,b	Bactericide	3.4 ± 0.9a,b	Bactericide
Calung	5.6 ± 0.7a	Bacteriostatic	7.0 ± 1.9b	Bacteriostatic	7.5 ± 1.1b	Bactericide	6.2 ± 2.0b	Bacteriostatic
Makin	4.1 ± 0.9a	Bacteriostatic	2.5 ± 0.5a	Bactericide	3.3 ± 0.3a	Bactericide	2.6 ± 0.6a	Bactericide
Kruet Mameh	4.3 ± 0.9a	Bactericide	4.1 ± 1.0a,b	Bactericide	6.8 ± 2.5a,b	Bacteriostatic	4.7 ± 1.5a,b	Bacteriostatic
Mentui	11.6 ± 3.5b	Bacteriostatic	4.8 ± 1.8a,b	Bacteriostatic	6.3 ± 1.0a,b	Bacteriostatic	4.1 ± 0.5a,b	Bacteriostatic
